# Antibiotic Use in the Emergency Department: A Retrospective Study in Indonesia

**DOI:** 10.3390/antibiotics15040401

**Published:** 2026-04-15

**Authors:** Ikhwan Yuda Kusuma, Ria Benkő, Erika Piroska Papfalvi, Ni Made Amelia Ratnata Dewi, Fiqih Nurkholis, Róbert Nacsa, Dezső Csupor, Mária Matuz

**Affiliations:** 1 Pharmacy Study Program, Faculty of Health, Universitas Harapan Bangsa, Purwokerto 53182, Indonesia; fiqihnurkholis@uhb.ac.id; 2 Institute of Clinical Pharmacy, Faculty of Pharmacy, University of Szeged, 6725 Szeged, Hungary; papfalvi.erika.piroska@med.u-szeged.hu (E.P.P.); ameliadewi@unram.ac.id (N.M.A.R.D.); csupor.dezso@szte.hu (D.C.); matuz.maria@szte.hu (M.M.); 3 Institute of Clinical Pharmacy, Albert Szent-Györgyi Health Centre, University of Szeged, 6725 Szeged, Hungary; 4 Emergency Department, Albert Szent-Györgyi Health Centre, University of Szeged, 6725 Szeged, Hungary; 5 Pharmacy Study Program, Faculty of Medicine and Health Sciences, University of Mataram, Mataram 83115, Indonesia; 6 Central Pharmacy, Cilacap Regional General Hospital, Cilacap 53223, Indonesia

**Keywords:** antimicrobial resistance, antimicrobial stewardship, emergency departments, Indonesia

## Abstract

**Background:** Antimicrobial resistance (AMR) is a global health threat arising from inappropriate antibiotic use. Data on the prescription of antibiotics in emergency departments (EDs), critical care points for infection management, are limited. **Objective:** This study aimed to assess systemic antibiotic use in an Indonesian ED. **Methods:** This retrospective observational study was conducted in the Cilacap Teaching Hospital ED in 2022. Data, including patient demographics and systemic antibiotic prescription details (World Health Organization Anatomical Therapeutic Chemical (WHO ATC): J01) were extracted from electronic medical records. Antibiotic use was analyzed according to age groups (children [0–14 years], adults [15–64 years], and the elderly [≥65 years]), administration route, and the World Health Organization Access, Watch, and Reserve classification. **Results:** Among all ED visits during the study period, 52.1% (14,396/27,640) received systemic antibiotics, and adults comprised 68.5% (9861/14,396) of antibiotic-exposed cases. Cephalosporins were the most frequently prescribed antibiotics in all age groups (42.4–50.9%). Penicillins were more frequently prescribed in children (29.9%) than in adults (10.0%) and the elderly (6.6%), whereas fluoroquinolones were more commonly prescribed in the elderly (21.1%) than in adults (16.2%) and children (3.8%). Watch-class antibiotics, comprising 63.9% of all prescriptions, were commonly prescribed in the elderly (71.9%). Oral route was the predominant form (65.8%), particularly in children (76.5%). The most frequently prescribed antibiotics differed across age groups, with amoxicillin followed by cefixime in children, and cefixime followed by ceftriaxone in both adults and the elderly. **Conclusions:** This study showed high antibiotic exposure and identified age-related differences in antibiotic prescribing, and patterns that warrant further evaluation within antimicrobial stewardship frameworks, to optimize antibiotic use and mitigate AMR.

## 1. Introduction

Antimicrobial resistance (AMR) is a critical global health challenge that threatens antibiotic efficacy and infectious disease management [1]. Predictive statistical models estimate that bacterial resistance was associated with approximately 4.95 million deaths worldwide in 2019 [2]. In the absence of decisive action, AMR-related deaths are predicted to dramatically increase, reaching 10 million deaths annually by 2050, surpassing the number of deaths due to other diseases [3]. The main drivers of AMR are overuse, misuse, and inappropriate prescribing of antibiotics [4]. Optimizing antibiotic use is essential to prevent the worsening of AMR.

Emergency departments (EDs) are important for managing a diverse range of infections and serve as an interface between outpatient and inpatient care [5]. Emergency physicians work under significant time pressure [6] and, unlike general practitioners, often have to reach prescribing decisions based on limited patient history. High patient turnover, staffing variability, and lack of continuity of care present further challenges to antibiotic stewardship [7]. Additionally, out-of-hours ED services are associated with a lower threshold for antibiotic initiation and suboptimal antibiotic choices [8]. Most ED-initiated antibiotic treatments are empirical, highlighting the importance of knowledge regarding local resistance patterns for effective antimicrobial stewardship [5]. Despite the significant burden of infectious diseases managed in the ED, data from Indonesian emergency department settings remain limited.

The present study aimed to quantify the prevalence, prescribing patterns, AWaRe distribution, and routes of administration of systemic antibiotics prescribed in the emergency department of a teaching hospital in Indonesia in 2022.

## 2. Results

### 2.1. Study Cohort and Antibiotic Use

Among 27,640 emergency department (ED) encounters, 14,396 (52.08%; 95% CI 51.49–52.67) involved exposure to at least one systemic antibiotic (see Table 1). The prevalence of antibiotic exposure differed across age groups, being highest among active adults (55.23%; 95% CI 54.50–55.96), followed by children (46.86%; 95% CI 45.36–48.37) and the elderly (45.94%; 95% CI 44.64–47.26). This difference was statistically significant (chi-square *p* < 0.001), although the effect size was small (Cramér’s V = 0.085), suggesting a weak association between age group and antibiotic exposure.

### 2.2. Antibiotic Prescribing Patterns Across Age Groups

Comparative analyses across age groups are presented in Table 2. While several differences across age groups were statistically significant after Benjamini–Hochberg adjustment, most effect sizes were very weak to moderate, indicating limited practical differences despite statistical significance. The distribution of antibiotic subclasses differed significantly across age groups, with several categories remaining non-significant after adjustment. Cephalosporins (J01D) were the most frequently prescribed subclass overall (49.6%). Penicillins (J01C) were more commonly prescribed in children (29.9%) compared with adults (10.0%) and the elderly (6.6%) (Cramér’s V = 0.221), whereas fluoroquinolones (J01M) were more frequently prescribed in adults (16.2%) and the elderly (21.1%) than in children (3.8%) (Cramér’s V = 0.138). For AWaRe classification, Watch-class antibiotics predominated across all age groups (63.9%), with the highest proportion observed in the elderly (71.9%), followed by adults (64.1%) and children (52.4%) (*p* < 0.001; Cramér’s V = 0.114). Access-class antibiotic use remained low across all age groups (26.3%), with only modest variation (Cramér’s V = 0.055).

The route of administration also differed significantly across age groups (*p* < 0.001), with parenteral use increasing with age (23.5% in children vs. 45.0% in the elderly; Cramér’s V = 0.128). Oral administration was more common in children (76.5%) compared with adults (66.4%) and the elderly (55.0%).

Temporal patterns of prescribing across days of the week did not differ significantly across age groups after Benjamini–Hochberg adjustment. Although not statistically significant, prescribing peaked on Wednesdays (18.1%) and was lowest on Sundays (5.2%). Detailed distributions of antibiotic subclasses followed by specific active agents, AWaRe categories, route of administration, and weekday prescribing patterns stratified by age group and sex are presented in Table 3.

### 2.3. Top Prescribed Antibiotics

As shown in Table 4, cefixime (20.0%, n = 2877) was the most frequently prescribed antibiotic in the overall study cohort, followed by ceftriaxone (12.9%, n =1857). In children, amoxicillin was the most frequently prescribed antibiotic (23.5%, n = 465), followed by cefixime (18.4%, n = 364). In adults, cefixime was the most frequently prescribed antibiotic (20.8%, n = 2050), followed by ceftriaxone (12.8%, n = 1264). In the elderly, cefixime was the most frequently prescribed antibiotic (18.1%, n = 463), followed by ceftriaxone (17.6%, n = 451).

### 2.4. Factors Associated with Antibiotic Selection

To further evaluate factors associated with antibiotic selection, binary logistic regression analyses were performed (Table 5). For Access-class antibiotics, both adults (OR 1.44; 95% CI 1.28–1.62; *p* < 0.001) and elderly patients (OR 1.21; 95% CI 1.05–1.39; *p* = 0.009) had higher odds of receiving Access antibiotics compared with children. For Watch-class antibiotics, the association with age group was stronger, particularly among elderly patients (OR 2.33; 95% CI 2.06–2.64; *p* < 0.001), followed by adults (OR 1.65; 95% CI 1.49–1.82; *p* < 0.001). Female sex was associated with slightly lower odds of receiving Watch-class antibiotics (OR 0.93; 95% CI 0.86–0.99; *p* = 0.028). Associations with day of the week were modest and not consistently observed across categories, although increased odds of Access-class prescribing were identified on Fridays (OR 1.25; 95% CI 1.09–1.42; *p* = 0.001) and Saturdays (OR 1.17; 95% CI 1.02–1.33; *p* = 0.027).

## 3. Discussion

To the best of our knowledge, this is the first study that comprehensively examined the scale and pattern of systemic antibiotic use in the ED of an Indonesian teaching hospital after the coronavirus disease 2019 (COVID-19) pandemic in 2022.

### 3.1. Demographic Characteristics

First, our analyses revealed that adult patients comprised the largest age group that was prescribed systemic antibiotics during the study period, and the prevalence of antibiotic use was highest among adults.

The higher proportion of antibiotic use observed among the elderly male patients might be hypothesized to be partly associated with the smoking behavior of Indonesian males [9]. According to the Indonesia Statistic Authority, in 2021, the prevalence of smoking was 56.36% among men aged 15–64 years, compared with 2.6% among women in the same age group [10]. The mortality rate due to smoking-related conditions in Indonesia, such as chronic obstructive pulmonary disease, has significantly increased between 2007 and 2017 [11]. In 2023, over 21.7% of elderly Indonesians smoked daily, with more than 41% experiencing related health issues [12]. Smoking is associated with increased susceptibility to respiratory infections and impaired immune function [13,14], which may contribute to a greater need for antibiotic treatment. These factors may partly explain the observed sex-related differences in antibiotic exposure; however, this interpretation should be considered hypothesis-generating, as smoking-related variables were not directly assessed in the present study. The lack of comprehensive tobacco regulation, such as Indonesia’s nonratification of the WHO Framework Convention on Tobacco Control, has exacerbated the burden of smoking-related infections in Indonesia [15].

### 3.2. Route of Administration

In all age groups, oral antibiotics were generally preferred due to convenience in outpatient settings. Solid oral formulations, such as tablets and capsules, are favored for their longer shelf life, ease of dosage adjustment, and practicality [16]. Tablets were the most commonly prescribed formulation in all age groups, including children. However, despite their wide availability, liquid formulations for some medicines, such as amoxicillin, were prescribed in only 27.5% pediatric cases. These practices underscore the challenges associated with ensuring appropriate formulations for pediatric patients. A study in Sri Lanka has recommended that healthcare providers should prioritize liquid formulations for young children, as solid formulations, including tablets and capsules, often require manipulation, such as crushing and splitting, which can lead to inaccurate dosing and reduced safety [17]. These recommendations are relevant for improving medication safety and adherence among children younger than 5 years of age.

The higher use of parenteral antibiotics observed in elderly patients likely reflects the clinical complexity and acuity (higher case mix) rather than route-related advantages commonly encountered in this population in the emergency department. Older adults more frequently present with severe infections and impaired oral intake [18], or conditions requiring a rapid therapeutic effect, which may justify initial parenteral therapy [19], which may lead clinicians to prefer parenteral therapy in acute care settings, although oral administration remains feasible in many cases.

Patterns of Antibiotic Utilization and AWaRe Classification revealed distinct age-related prescribing patterns for beta-lactam antibiotics. Cefixime, a third-generation cephalosporin, was the most prescribed antibiotic in the overall cohort, in adults and in the elderly, whereas the penicillin derivative, amoxicillin, was the most frequently prescribed in children. Ceftriaxone, another third-generation cephalosporin, was the second most commonly prescribed antibiotic in the elderly and in adults, indicating a preference for the prescription of parenteral cephalosporins in older patients. These findings are consistent with previous Indonesian hospital-based studies, in which ceftriaxone, cefixime, and levofloxacin were among the most frequently used antibiotics in urinary tract infections [20] and community-acquired pneumonia management [21].

Indonesia’s national pharmaceutical sales data for 2000–2015 reveal that increased antibiotic consumption increased over this period, particularly for broad-spectrum penicillins (2.6-fold), fluoroquinolones (7.1-fold), and cephalosporins (5.1-fold) [22]. In 2021, Indonesia’s total antibiotic consumption was 1,183,877,975 defined daily doses, increasing to 1,430,286,555 defined daily doses in 2022 [23]. Our findings indicate that broad-spectrum beta-lactams, particularly third-generation cephalosporins, remain widely prescribed antibiotics across all age groups in Indonesia, despite their classification as Watch-class antibiotics. Amoxicillin, a narrow-spectrum penicillin, was among the most commonly prescribed antibiotics in our study, consistent with previous findings indicating its widespread use in outpatient settings [24]. Amoxicillin remains the first-line treatment for community-acquired pneumonia in children because of its efficacy against Streptococcus pneumoniae [25]. Additionally, amoxicillin is the preferred treatment for nonallergic patients with pharyngitis, as recommended by the Infectious Diseases Society of America [26], and a primary choice for acute bacterial sinusitis [27].

Comparable emergency department studies remain relatively limited, particularly from Indonesia [28] and other low- and middle-income settings [29,30]. In addition, ED-specific studies reporting detailed route-of-administration patterns are scarce, which limits direct comparison of this aspect across settings. Nevertheless, the available evidence shows several consistent patterns. In Indonesia [28], a retrospective study from an emergency department in Semarang reported that oral antibiotics predominated (76.09%), with ciprofloxacin, amoxicillin, and cefixime among the most frequently prescribed agents, which is broadly consistent with the high use of oral antibiotics and high usage of cefixime observed in the present study. Study in Sri Lanka [30], antibiotic utilization in an emergency treatment unit was characterized by frequent antibiotic use, particularly for respiratory tract infections, with amoxicillin/clavulanic acid and clarithromycin among the most common agents [26,27]. In Malaysia [29], higher antibiotic use was reported during weekend ED visits, whereas in the present study, prescribing peaked on Wednesdays and was lowest on Sundays, which may reflect lower patient volume or differences in case-mix during weekends, although these factors were not directly assessed in the present study, suggesting that temporal prescribing patterns may vary across ED settings. In Bahrain [31] and Australia [32], ED-based studies focused on prescribing appropriateness rather than overall utilization, but both emphasized the importance of antimicrobial stewardship because inappropriate antibiotic prescribing remained common. Similar concerns were also reported in pediatric emergency settings in Iran [33] and in emergency departments in South Africa [34], where broad-spectrum antibiotic use and lack of guideline adherence were important stewardship issues. In addition, a recent review from Latin America [35] highlighted that antimicrobial stewardship efforts in pediatric emergency departments remain limited, reinforcing the need for more ED-specific evidence from diverse healthcare settings.

During the initial phase of the COVID-19 pandemic, azithromycin was used due to its presumed immunomodulatory and antiviral properties [36]. However, its administration was subsequently restricted to patients with confirmed bacterial coinfections in light of emerging evidence [37]. The WHO has advised against the use of azithromycin for COVID-19 treatment due to concerns regarding potential cardiotoxicity and the increased risk of AMR [38]. To the best of our knowledge, no ED-specific Indonesian study has reported azithromycin utilization during the COVID-19 period in a directly comparable manner. However, hospital-based Indonesian studies indicate that azithromycin was used extensively during the early pandemic. In a multicenter retrospective cohort of hospitalized COVID-19 patients in Indonesia, chloroquine/hydroxychloroquine plus azithromycin was the most common treatment regimen (79.4%) [39]. Similarly, in a public hospital cohort from Bengkulu, Indonesia, 88.8% of pre-vaccinated COVID-19 pneumonia patients received azithromycin in combination with oseltamivir [40]. In contrast, azithromycin accounted for 10.3% of all antibiotic prescriptions in the present post-pandemic ED cohort, suggesting substantially lower and more selective use than during the pandemic period. This difference should be interpreted cautiously, as the Indonesian comparator studies involved hospitalized COVID-19 patients during the early pandemic, whereas the present study reflects post-pandemic antibiotic prescribing in a general ED population. In contrast, azithromycin accounted for 10.3% of all antibiotic prescriptions in the present post-pandemic ED cohort, suggesting more selective use compared with reports from the early COVID-19 period. However, direct comparison of exposure rates is limited due to differences in study populations and settings. This interpretation is further supported by safety concerns reported in Indonesian hospitals, including QT prolongation associated with azithromycin-containing COVID-19 regimens [41]. Several drug usage studies from Hungary [42], Bosnia and Herzegovina [43], and Spain [44] also reported increased use of macrolides, particularly azithromycin, in ambulatory care settings during and after the COVID-19 pandemic.

Fluoroquinolones, particularly levofloxacin, were more frequently prescribed in the elderly than in adults, likely because of their broad-spectrum efficacy against common infections observed in older populations, such as urinary tract and respiratory tract infections and bacterial gastroenteritis. In elderly patients, ceftriaxone, levofloxacin, and gentamicin were the most frequently prescribed antibiotics. The aminoglycoside gentamicin is commonly prescribed for severe infections, such as sepsis and complicated urinary tract infections, which are more prevalent among older adults with comorbidities [45]. However, aminoglycosides may be ototoxic and nephrotoxic, raising concerns due to age-related decline in renal function [46]. Fluoroquinolones, such as levofloxacin, which are widely prescribed in this population, should be used with caution due to age-related pharmacokinetic changes that increase the risk of adverse effects [47]. Additionally, fluoroquinolones increase the risk of *Clostridioides difficile* infection [48] and prolong the QT interval, raising the risk of *torsades de pointes*, especially in elderly patients [49]. These safety considerations are important for contextualizing the observed prescribing patterns and should be interpreted alongside findings from existing international studies on antibiotic use in emergency care settings, although directly comparable ED-specific data remain limited.

### 3.3. AWaRe Classification and Stewardship Implications

This study also observed that the Watch-class drugs were the most frequently prescribed antibiotics, highlighting a significant area for future interventions to improve antibiotic stewardship [50]. The WHO recommends that at least 60% of the prescribed antibiotics should be from the Access class to minimize AMR risk [51]. Similarly, the European Center for Disease Prevention and Control has set a target of 65% for the consumption of Access-class antibiotics by 2030. Although in 2022, only 36% of the EU/EEA countries met this threshold, with an average Access-class antibiotic usage of 59.8% [52]. In the present study, only 26.3% of the antibiotic prescriptions fell into the Access class, which is substantially below the WHO-recommended target of at least 60% Access-class use, indicating consistently low use and a marked deviation from global antimicrobial stewardship benchmarks.

An age-related pattern was observed in the multivariable analysis, with higher odds of both Access- and Watch-class prescribing among adults and elderly patients compared with children. Specifically, elderly patients had the highest odds of receiving Watch-class antibiotics (OR 2.33; 95% CI 2.06–2.64; *p* < 0.001), followed by adults (OR 1.65; 95% CI 1.49–1.82; *p* < 0.001). Female sex was associated with slightly lower odds of Watch-class prescribing (OR 0.93; 95% CI 0.86–0.99; *p* = 0.028), whereas no significant association was observed for Access-class prescribing.

Several factors might have contributed to the high use of Watch-class antibiotics in the current study, including the prescribers’ preference for broader-spectrum agents for empiric treatment in emergency settings where rapid clinical decision-making is required. Antibiotic selection in emergency departments may also be influenced by local formulary availability and antimicrobial resistance patterns, which are recognized drivers of prescribing behavior in clinical practice [23], although such data were not available in the present study. Given that Watch-class antibiotics are associated with a higher risk of resistance, healthcare systems must prioritize the availability and use of Access-class antibiotics to minimize reliance on broad-spectrum agents [53,54]. These findings suggest the need for targeted antimicrobial stewardship strategies in the emergency department, including promoting guideline-based empiric prescribing, increasing the use of Access-class antibiotics where appropriate, and supporting periodic review of prescribing practices.

Interestingly, no Reserve-class antibiotics were prescribed at the ED setting in the present study. The Reserve class, designated for last-resort treatments in cases where other options fail, reflects adherence to stewardship protocols that preserve these agents for severe, treatment-resistant cases and may also reflect the limited routine availability of Reserve antibiotics in the ED [55]. Global data show that lower and upper middle-income countries tend to rely more on Watch-class antibiotics, and that the use of Access-class antibiotics is higher in high-income countries [53].

### 3.4. Temporal Patterns in Antibiotic Exposures

Finally, the daily distribution of antibiotic use patterns revealed that the highest and lowest rates of antibiotic prescriptions occurred on Wednesdays and Sundays, respectively, in the ED. However, after adjustment for multiple comparisons, these differences were not statistically significant, indicating that weekday variation was limited. This finding is in contrast with a Malaysian study, which reported that weekend ED visits were associated with a higher rate of antibiotic use, particularly in patients with upper respiratory tract infections and pediatric patients with prolonged fever [29]. In the present study, multivariable analysis suggested that weekday effects were generally modest and inconsistent. Taken together, these findings suggest that temporal variation in prescribing was minimal and likely influenced by factors such as patient case-mix and variations in clinical workload, although these variables were not directly captured in the present study.

### 3.5. Limitations

The present study has several limitations. First, our single-center design restricts the generalizability of our findings to other healthcare settings and regions. Second, although highlighting patterns in antibiotic use, we did not account for microbiologic or clinical outcomes, hindering our ability to provide deeper insights into the suitability of the prescribed antibiotics. Finally, although diagnostic codes were collected, diagnostic stratification was not performed. The recorded ICD codes were frequently non-specific or inconsistent, limiting reliable grouping into clinically meaningful categories. In addition, ED diagnoses often represent provisional or multiple working diagnoses that may be revised after further evaluation, which could introduce misclassification bias in stratified analyses.

## 4. Methodology

### 4.1. Study Design and Setting

This was a retrospective observational study including all emergency department (ED) visits recorded at Cilacap Teaching Hospital, Indonesia, between 1 January and 31 December 2022. The unit of analysis was the ED visit rather than unique patients; therefore, repeated visits by the same patient during the study period were treated as separate encounters, reflecting routine clinical practice and prescribing decisions in the ED. Cilacap Teaching Hospital, a 400-bed healthcare facility located in the Cilacap District, is the largest hospital in the most populous district of Central Java, Indonesia. The hospital provides regional-level healthcare to 2 million individuals. The ED of Cilacap Teaching Hospital has 19 beds and manages over 27,000 patients annually and approximately 360 visits/day. During peak hours (07:00–14:00), at least 2–4 emergency physicians are on duty, whereas 2–3 emergency physicians remain available to manage critical cases during the afternoon and night shifts (14:00–21:00 and 21:00–07:00 respectively) on both weekdays and weekends. Emergency protocols are in place to manage severe and life-threatening emergencies, such as acute coronary syndrome, ischemic stroke, sepsis, anaphylactic shock, and major trauma, during all hours. These protocols involve nurses trained in advanced life support and senior emergency physicians to ensure timely and appropriate care for critical cases. Patients in the ED predominantly comprise adults with acute medical, surgical, and trauma emergencies.

### 4.2. Data Collection

**Figure 1:**
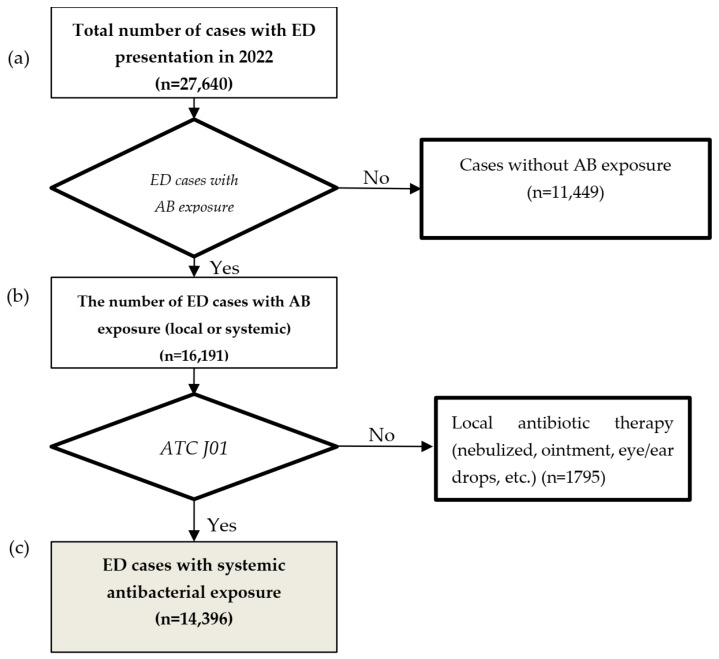
Study flowchart. Note: (a) total ED cases; (b) ED cases with any antibacterial exposure; (c) ED cases with systemic antibacterial exposure; ATC, anatomical therapeutic chemical; J01, antibacterials for systemic use; AB, antibiotics.

Data were obtained from the electronic medical records stored in the hospital management information system of Cilacap Teaching Hospital. Collected data were entered into a Microsoft Excel spreadsheet generated for this purpose. Variables captured for the present study included presentation and discharge dates; patient age and sex; diagnosis based on the International Classification of Diseases code; and name, dose, dosage form, dosing schedule/frequency, and route of administration of the antibiotics. The data of all patients who presented to the emergency department (ED) during the study period were screened to identify ED encounters with any antibacterial exposure (local or systemic). Subsequently, cases with at least one systemic antibiotic in the J01 class (antibacterials for systemic use) according to the World Health Organization Anatomical Therapeutic Chemical (WHO ATC) classification system were retained for analysis (Figure 1), regardless of whether the antibiotic was administered in the ED, prescribed at discharge, or continued during hospitalization. Antibiotic exposure was defined at the ED encounter level; each ED visit was classified as antibiotic-exposed if at least one systemic antibiotic was administered during the ED stay and/or prescribed at discharge. When multiple antibiotics (including different agents administered in the ED and prescribed at discharge) were recorded within the same encounter, they were considered part of a single exposure event at the encounter level, while individual antibiotics were retained for drug-level analyses. Systemic antibiotics were further categorized according to the World Health Organization Access, Watch, and Reserve (AWaRe) classification (WHO AWaRe 2023 version). Antifungals, antivirals, antituberculosis, and antiparasitic as well as local antibacterial therapies were excluded. Data on monthly and annual patient turnover were derived from the hospital pharmacy administrative records.

### 4.3. Statistical Analysis

The number and percentage of ED encounters with systemic antibiotic-exposure were calculated, and the prevalence estimates were reported with 95% confidence intervals (95% CIs). Additionally, data were stratified by age group, wherein children, adults, and the elderly were defined as those aged 0–14, 15–64, and ≥65 years, respectively, and the top 10 antibiotics prescribed for each age group were identified. Categorical variables were compared across age groups using the chi-squared test. To account for multiple comparisons, *p*-values were adjusted using the Benjamini–Hochberg procedure to control the false discovery rate. Alongside the chi-squared test, Cramér’s V was calculated to quantify effect size, with values < 0.1 interpreted as very weak associations. In addition, binary logistic regression analyses were performed to assess factors associated with prescribing Access-class and Watch-class antibiotics. Sex, age group, and day of the week were entered as independent variables. Results are presented as odds ratios (ORs) with 95% confidence intervals, where the OR was used as the measure of effect size. Descriptive statistics and statistical analyses were performed using Microsoft Excel and SPSS 29.0.

## 5. Conclusions

The present study demonstrated that systemic antibiotic exposure in the emergency department was high, affecting over half of all ED encounters, with clear age-related differences in prescribing patterns. Broad-spectrum antibiotics, particularly Watch-class antibiotics, predominated across all age groups, whereas Access-class use remained consistently low. These findings underscore the need for targeted antimicrobial stewardship strategies to optimize antibiotic selection and reduce reliance on broad-spectrum agents in emergency care settings.

## Figures and Tables

**Table 1:** Systemic antibiotic exposure by age group.

	N	Received Systemic Antibiotics	% (95% CI)	*p*	Effect Size (Cramér V)
Children	4225	1980	46.86% (95% CI: 45.36–48.37)	*p* < 0.001 *	0.08527
Active Adults	17,854	9861	55.23% (95% CI: 54.5–55.96)		
Elderly	5561	2555	45.94% (95% CI: 44.64–47.26)		
Total	27,640	14,396	52.08% (95% CI: 51.49–52.67)		

* Chi-squared test.

**Table 2:** Antibiotic prescribing patterns by age group.

		Children	Active Adults	Elderly	Total	Effect Size (Cramér V)	*p* *
		0–14	15–64	65+			
	N	1980	9861	2555	14,396		
Sex	Female	706 (35.7%)	4864 (49.3%)	964 (37.7%)	6534 (45.4%)	0.117	<0.001
	Male	1274 (64.3%)	4997 (50.7%)	1591 (62.3%)	7862 (54.6%)	0.117	<0.001
Antibiotic Subclass (ATC)	Tetracyclines (J01A)	2 (0.1%)	23 (0.2%)	5 (0.2%)	30 (0.2%)	0.01	0.494
	Amphenicols (J01B)	9 (0.5%)	29 (0.3%)	3 (0.1%)	41 (0.3%)	0.018	0.115
	Beta-Lactam Antibacterials, Penicillins (J01C)	592 (29.9%)	982 (10%)	169 (6.6%)	1743 (12.1%)	0.221	<0.001
	Other Beta-Lactam Antibacterials (J01D)	841 (42.5%)	5016 (50.9%)	1285 (50.3%)	7142 (49.6%)	0.057	<0.001
	Sulfonamides and Trimethoprim (J01E)	34 (1.7%)	374 (3.8%)	20 (0.8%)	428 (3%)	0.073	<0.001
	Macrolides, Lincosamides and Streptogramins (J01F)	365 (18.4%)	1342 (13.6%)	325 (12.7%)	2032 (14.1%)	0.05	<0.001
	Aminoglycoside Antibacterials (J01G)	49 (2.5%)	213 (2.2%)	161 (6.3%)	423 (2.9%)	0.093	<0.001
	Quinolone Antibacterials (J01M)	75 (3.8%)	1594 (16.2%)	539 (21.1%)	2208 (15.3%)	0.138	<0.001
	Other Antibacterials (J01X)	13 (0.7%)	288 (2.9%)	48 (1.9%)	349 (2.4%)	0.052	<0.001
Antibiotic AWaRe	Access	418 (21.1%)	2742 (27.8%)	622 (24.3%)	3782 (26.3%)	0.055	<0.001
	Watch	1037 (52.4%)	6325 (64.1%)	1838 (71.9%)	9200 (63.9%)	0.114	<0.001
	Reserved	0 (0%)	0 (0%)	0 (0%)	0 (0%)	NA	NA
	Not Listed	525 (26.5%)	794 (8.1%)	95 (3.7%)	1414 (9.8%)	0.231	<0.001
Administration	Parenteral	465 (23.5%)	3310 (33.6%)	1151 (45%)	4926 (34.2%)	0.128	<0.001
	Oral	1515 (76.5%)	6551 (66.4%)	1404 (55%)	9470 (65.8%)	0.128	<0.001
	Liquid Oral	501 (25.3%)	7 (0.1%)	1 (0%)	509 (3.5%)	NA	NA
	Solid Oral	1014 (51.2%)	6544 (66.4%)	1403 (54.9%)	8961 (62.2%)	NA	NA
Days	Sunday	122 (6.2%)	489 (5%)	141 (5.5%)	752 (5.2%)	0.019	0.16
	Monday	342 (17.3%)	1584 (16.1%)	457 (17.9%)	2383 (16.6%)	0.02	0.16
	Tuesday	329 (16.6%)	1636 (16.6%)	373 (14.6%)	2338 (16.2%)	0.021	0.16
	Wednesday	347 (17.5%)	1769 (17.9%)	485 (19%)	2601 (18.1%)	0.012	0.529
	Thursday	296 (14.9%)	1602 (16.2%)	410 (16%)	2308 (16%)	0.012	0.529
	Friday	290 (14.6%)	1436 (14.6%)	355 (13.9%)	2081 (14.5%)	0.007	0.67
	Saturday	254 (12.8%)	1345 (13.6%)	334 (13.1%)	1933 (13.4%)	0.009	0.618

Note: ATC, Anatomical Therapeutic Chemical classification; N, number of prescriptions; %, percentage; NA, not applicable. * chi-square test; Benjamini–Hochberg adjusted.

**Table 3:** Antibiotic subclasses (ATC), AWaRe classification, route of administration, and weekday prescribing patterns stratified by age group and sex.

		Children		Active Adults		Elderly		
		0–14 Years		15–64 Years		65+ Years		
		Female	Male	Female	Male	Female	Male	Total
	N	706	1274	4864	4997	964	1591	14,396
Antibiotic Subclass (ATC)	Tetracyclines (J01A)	0 (0.0%)	2 (0.2%)	6 (0.1%)	17 (0.3%)	5 (0.5%)	0 (0.0%)	30 (0.2%)
	Amphenicols (J01B)	2 (0.3%)	7 (0.5%)	21 (0.4%)	8 (0.2%)	1 (0.1%)	2 (0.1%)	41 (0.3%)
	Beta-Lactam Antibacterials, Penicillin (J01C)	230 (32.6%)	362 (28.4%)	600 (12.3%)	382 (7.6%)	62 (6.4%)	107 (6.7%)	1743 (12.1%)
	Other Beta-Lactam Antibacterials (J01D)	261 (37%)	580 (45.5%)	2335 (48%)	2681 (53.7%)	427 (44.3%)	858 (53.9%)	7142 (49.6%)
	Sulfonamides and Trimethoprim (J01E)	16 (2.3%)	18 (1.4%)	101 (2.1%)	273 (5.5%)	5 (0.5%)	15 (0.9%)	428 (3%)
	Macrolides, Lacosamide and Streptogramins (J01F)	143 (20.3%)	222 (17.4%)	801 (16.5%)	541 (10.8%)	160 (16.6%)	165 (10.4%)	2032 (14.1%)
	Aminoglycoside Antibacterials (J01G)	21 (3%)	28 (2.2%)	101 (2.1%)	112 (2.2%)	75 (7.8%)	86 (5.4%)	423 (2.9%)
	Quinolone Antibacterials (J01M)	30 (4.2%)	45 (3.5%)	755 (15.5%)	839 (16.8%)	211 (21.9%)	328 (20.6%)	2208 (15.3%)
	Other Antibacterials (J01X)	3 (0.4%)	10 (0.8%)	144 (3%)	144 (2.9%)	18 (1.9%)	30 (1.9%)	349 (2.4%)
Antibiotic agent	Ampicillin (J01CA01)	26 (3.7%)	33 (2.6%)	0 (0%)	1 (0%)	0 (0%)	0 (0%)	60 (0.4%)
	Amoxicillin (J01CA04)	161 (22.8%)	304 (23.9%)	429 (8.8%)	241 (4.8%)	20 (2.1%)	41 (2.6%)	1196 (8.3%)
	Phenoxymethylpenicillin (J01CE02)	0 (0.0%)	0 (0.0%)	14 (0.3%)	0 (0.0%)	0 (0.0%)	0 (0.0%)	14 (0.1%)
	Benzathine benzylpenicillin (J01CE08)	1 (0.1%)	1 (0.1%)	4 (0.1%)	4 (0.1%)	0 (0.0%)	0 (0.0%)	10 (0.1%)
	Ampicillin and beta-lactamase inhibitor (J01CR01) Parenteral	22 (3.1%)	15 (1.2%)	12 (0.2%)	25 (0.5%)	7 (0.7%)	8 (0.5%)	89 (0.6%)
	ampicillin and beta-lactamase inhibitor (J01CR02) Oral	20 (2.8%)	9 (0.7%)	141 (2.9%)	111 (2.2%)	35 (3.6%)	58 (3.6%)	374 (2.6%)
	Cefazolin (J01DB04)	11 (1.6%)	34 (2.7%)	337 (6.9%)	351 (7%)	38 (3.9%)	123 (7.7%)	894 (6.2%)
	Cefadroxil (J01DB05)	49 (6.9%)	133 (10.4%)	312 (6.4%)	272 (5.4%)	44 (4.6%)	46 (2.9%)	856 (5.9%)
	Cefotaxime (J01DD01)	24 (3.4%)	42 (3.3%)	42 (0.9%)	19 (0.4%)	1 (0.1%)	6 (0.4%)	134 (0.9%)
	Ceftazidime (J01DD02)	0 (0%)	0 (0%)	20 (0.4%)	27 (0.5%)	8 (0.8%)	24 (1.5%)	79 (0.5%)
	Ceftriaxone (J01DD04)	58 (8.2%)	84 (6.6%)	537 (11%)	727 (14.5%)	103 (10.7%)	348 (21.9%)	1857 (12.9%)
	Ceftizoxime (J01DD07)	5 (0.7%)	37 (2.9%)	110 (2.3%)	170 (3.4%)	49 (5.1%)	25 (1.6%)	396 (2.8%)
	Cefixime (J01DD08)	114 (16.1%)	250 (19.6%)	962 (19.8%)	1088 (21.8%)	181 (18.8%)	282 (17.7%)	2877 (20%)
	Cefoperazone and beta-lactamase inhibitor (J01DD62)	0 (0.0%)	0 (0.0%)	9 (0.2%)	19 (0.4%)	1 (0.1%)	4 (0.3%)	33 (0.2%)
	Meropenem (J01DH02)	0 (0.0%)	0 (0.0%)	6 (0.1%)	5 (0.1%)	2 (0.2%)	0 (0.0%)	13 (0.1%)
	Imipenem and cilastatin (J01DH51)	0 (0.0%)	0 (0.0%)	0 (0%)	3 (0.1%)	0 (0.0%)	0 (0.0%)	3 (0.1%)
Antibiotic AWaRe	Access	147 (20.8%)	271 (21.3%)	1335 (27.4%)	1407 (28.2%)	243 (25.2%)	379 (23.8%)	3782 (26.3%)
	Watch	372 (52.7%)	665 (52.2%)	3056 (62.8%)	3269 (65.4%)	694 (72%)	1144 (71.9%)	9200 (63.9%)
	Reserved	0 (0.0%)	0 (0.0%)	0 (0.0%)	0 (0.0%)	0 (0.0%)	0 (0.0%)	0 (0.0%)
	Not Listed	187 (26.5%)	338 (26.5%)	473 (9.7%)	321 (6.4%)	27 (2.8%)	68 (4.3%)	1414 (9.8%)
Administration	Parenteral	177 (25.1%)	288 (22.6%)	1500 (30.8%)	1810 (36.2%)	364 (37.8%)	787 (49.5%)	4926 (34.2%)
	Oral	529 (74.9%)	986 (77.4%)	3364 (69.2%)	3187 (63.8%)	600 (62.2%)	804 (50.5%)	9470 (65.8%)
	Liquid Oral	190 (26.9%)	311 (24.4%)	2 (0.1%)	5 (0.1%)	0 (0.0%)	1 (0.1%)	509 (3.5%)
	Solid Oral	339 (48%)	675 (53%)	3362 (69.1%)	3182 (63.7%)	600 (62.2%)	803 (50.5%)	8961 (62.2%)
Days	Sunday	34 (4.8%)	88 (6.9%)	235 (4.8%)	254 (5.1%)	42 (4.4%)	99 (6.2%)	752 (5.2%)
	Monday	111 (15.7%)	231 (18.1%)	783 (16.1%)	801 (16%)	201 (20.9%)	256 (16.1%)	2383 (16.6%)
	Tuesday	103 (14.6%)	226 (17.7%)	777 (16%)	859 (17.2%)	133 (13.8%)	240 (15.1%)	2338 (16.2%)
	Wednesday	143 (20.3%)	204 (16%)	881 (18.1%)	888 (17.8%)	180 (18.7%)	305 (19.2%)	2601 (18.1%)
	Thursday	114 (16.1%)	182 (14.3%)	807 (16.6%)	795 (15.9%)	155 (16.1%)	255 (16%)	2308 (16%)
	Friday	109 (15.4%)	181 (14.2%)	707 (14.5%)	729 (14.6%)	144 (14.9%)	211 (13.3%)	2081 (14.5%)
	Saturday	92 (13%)	162 (12.7%)	674 (13.9%)	671 (13.4%)	109 (11.3%)	225 (14.1%)	1933 (13.4%)

Note: ATC, Anatomical Therapeutic Chemical classification; AWaRe, Access, Watch, Reserve classification; N, number of prescriptions; %, percentage.

**Table 4:** The top 10 prescribed antibiotics in the three age groups.

All Age Group			Children			Adults			Elderly		
ATC Code	Active Agent	Total (%)	ATC Code	Active Agent	Total in Children (%)	ATC Code	Active Agent	Total in Adults (%)	ATC7	Active Agent	Total in Elderly (%)
J01DD08	cefixime	2877 (20.0%)	J01CA04	amoxicillin	465 (23.5%)	J01DD08	cefixime	2050 (20.8%)	J01DD08	cefixime	463 (18.1%)
J01DD04	ceftriaxone	1857 (12.9%)	J01DD08	cefixime	364 (18.4%)	J01DD04	ceftriaxone	1264 (12.8%)	J01DD04	ceftriaxone	451 (17.6%)
J01MA12	levofloxacin	1487 (10.3%)	J01FA10	azithromycin	240 (12.1%)	J01MA12	levofloxacin	1029 (10.4%)	J01MA12	levofloxacin	416 (16.3%)
J01FA10	azithromycin	1483 (10.3%)	J01DB05	cefadroxil	182 (9.2%)	J01FA10	azithromycin	956 (9.7%)	J01FA10	azithromycin	287 (11.2%)
J01CA04	amoxicillin	1196 (8.3%)	J01DD04	ceftriaxone	142 (7.2%)	J01DB04	cefazolin	688 (6.9%)	J01DB04	cefazolin	161 (6.3%)
J01DB04	cefazolin	894 (6.2%)	J01FA01	erythromycin	106 (5.4%)	J01CA04	amoxicillin	670 (6.8%)	J01GB03	gentamicin	161 (6.3%)
J01DB05	cefadroxil	856 (6.0%)	J01DD01	cefotaxime	66 (3.3%)	J01DB05	cefadroxil	584 (5.9%)	J01MA02	ciprofloxacin	99 (3.9%)
J01MA02	ciprofloxacin	625 (4.3%)	J01CA01	ampicillin	59 (3%)	J01MA02	ciprofloxacin	493 (5.0%)	J01CR02	AMC	93 (3.6%)
J01EE01	SMX/TMP	428 (3.0%)	J01GB03	gentamicin	47 (2.4%)	J01EE01	SMX/TMP	374 (3.8%)	J01DB05	cefadroxil	90 (3.5%)
J01GB03	gentamicin	419 (2.9%)	J01DB04	cefazolin	45 (2.3%)	J01XD01	metronidazole	288 (2.9%)	J01DD07	ceftizoxime	74 (2.9%)

Note: ATC, Anatomical Therapeutic Chemical classification; %, percentage; AMC, amoxicillin and beta-lactamase inhibitor; SMX/TMP, sulfamethoxazole and trimethoprim.

**Table 5:** Factors associated with Access and Watch antibiotic prescribing (logistic regression).

	Access Antibiotics			Watch Antibiotics		
Characteristic	OR	95% CI	*p*-Value	OR	95% CI	*p*-Value
**Sex**						
Male	—	—		—	—	
Female	0.98	0.91, 1.06	0.7	0.93	0.86, 0.99	0.028
**Age group**						
Children	—	—		—	—	
Adults	1.44	1.28, 1.62	<0.001	1.65	1.49, 1.82	<0.001
Elderly	1.21	1.05, 1.39	0.009	2.33	2.06, 2.64	<0.001
**Day of week**						
Monday	—	—		—	—	
Tuesday	1.06	0.93, 1.21	0.3	0.89	0.79, 1.00	0.057
Wednesday	1.01	0.89, 1.15	0.9	1.01	0.90, 1.14	0.8
Thursday	0.94	0.82, 1.07	0.3	0.99	0.88, 1.12	0.9
Friday	1.25	1.09, 1.42	0.001	0.82	0.72, 0.92	0.001
Saturday	1.17	1.02, 1.33	0.027	0.89	0.78, 1.01	0.066
Sunday	0.85	0.70, 1.04	0.12	0.97	0.81, 1.15	0.7

Note: CI = 95%; Confidence Interval; OR = Odds Ratio.

## Data Availability

The original contributions presented in the study are included in the article; further inquiries can be directed to the corresponding authors.
